# The Community Lab of Ideas for Health: Community-Based Transdisciplinary Solutions in a Malaria Elimination Trial in The Gambia

**DOI:** 10.3389/fpubh.2021.637714

**Published:** 2021-07-20

**Authors:** Yoriko Masunaga, Fatou Jaiteh, Ebrima Manneh, Julie Balen, Joseph Okebe, Umberto D'Alessandro, Claudia Nieto-Sanchez, Daniel H. de Vries, René Gerrets, Koen Peeters Grietens, Joan Muela Ribera

**Affiliations:** ^1^Department of Public Health, Unit of Socio-Ecological Health Research, Institute of Tropical Medicine, Antwerp, Belgium; ^2^Department of Sociology and Anthropology, Faculty of Social and Behavioural Science, University of Amsterdam, Amsterdam, Netherlands; ^3^Medical Research Council Unit The Gambia at the London School of Hygiene and Tropical Medicine, Fajara, Gambia; ^4^School of Health and Related Research, University of Sheffield, Sheffield, United Kingdom; ^5^Department of International Public Health, Liverpool School of Tropical Medicine, Liverpool, United Kingdom; ^6^School of Tropical Medicine and Global Health, Nagasaki University, Nagasaki, Japan; ^7^PASS Suisse, Neuchâtel, Switzerland; ^8^Medical Anthropology Research Centre (MARC) at Departament d'Antropologia, Filosofia i Treball Social, Universitat Rovira i Virgili, Tarragona, Spain

**Keywords:** The Gambia, dialogical approach, living lab, co-creation, transdisciplinary research, participatory approach, community-based solutions, malaria elimination program

## Abstract

**Background:** Community participation in global health interventions may improve outcomes and solve complex health issues. Although numerous community participatory approaches have been developed and introduced, there has been little focus on “how” and “who” to involve in the implementation of community-based clinical trials where unequal distribution of power between implementers and communities pre-exists. Addressing *how* to achieve community-based solutions in a malaria elimination trial in The Gambia, we developed the Community Lab of Ideas for Health (CLIH): a participatory approach that enabled communities to shape trial implementation.

**Methods:** As part of transdisciplinary research, we conducted qualitative research with in-depth interviews, discussions, and observations in 17 villages in the North Bank Region of The Gambia between March 2016 and December 2017. We designed an iterative research process involving ethnography, stakeholder-analysis, participatory-discussions, and qualitative monitoring and evaluation, whereby each step guided the next. We drew upon ethnographic results and stakeholder-analysis to identify key-informants who became participants in study design and implementation. The participatory-discussions provided a co-creative space for sharing community-centric ideas to tackle trial implementation challenges. The proposed strategies for trial implementation were continuously refined and improved through our monitoring and evaluation.

**Results:** The CLIH incorporated communities' insights, to co-create tailored trial implementation strategies including: village health workers prescribing and distributing antimalarial treatments; “compounds” as community-accepted treatment units; medicine distribution following compound micro-politics; and appropriate modes of health message delivery. Throughout the iterative research process, the researchers and communities set the common goal, namely to curtail the *medical poverty trap* by reducing malaria transmission and the burden thereof. This innovative collaborative process built trust among stakeholders and fully engaged researchers and communities in co-creation and co-implementation of the trial.

**Discussion:** The CLIH approach succeeded in touching the local realities by incorporating a spectrum of perspectives from community-members and discerning project-derived knowledge from local-knowledge. This process allowed us to co-develop locally-oriented solutions and ultimately to co-establish an intervention structure that community-members were ready and willing to use, which resulted in high uptake of the intervention (92% adherence to treatment). Successfully, the CLIH contributed in bridging research and implementation.

## Introduction

There is longstanding interest among health professionals and researchers in involving local communities to find solutions to complex health issues (1). The World Health Organization (WHO) recommends and promotes community participation in disease control (2) as a way to improve the outcomes of health interventions (3). Over the past few decades, there has been a shift in the public health definition of communities from “research-targets” to “counterparts” in an effort to alter the power-dynamics between researchers and local populations (4–6). Many community participatory approaches have attempted to address the disparities and inequities in health interventions, with researchers and communities striving to contribute equally at all phases of the research process, integrate knowledge and action, and facilitate co-learning and empowering processes, which are believed to increase a community's control over its social environment (1, 4). The presumed role of communities has become that of decision-making participants who generate and provide community-influenced/based knowledge in a research process (e.g. problem definition, data collection and analysis) (4, 7).

Community participation and community-based solutions, however, can be an overused buzzword (8, 9). Given the challenges in overcoming the disparities between researchers and communities, equal contribution to “all phases” of the research process has remained elusive. “Community participation” is regularly claimed as a tokenistic and instrumental way of legitimizing interventions by increasing community visibility (8, 10–14) whereas, in reality, community involvement is limited to a passive role in the decision-making process (11, 12, 14). In settings such as clinical trials, a research/trial framework is often pre-set (e.g. the development of topics and goals, budgeting, selecting communities), with researchers and trial implementers privy to specific knowledge and expertise that is not necessarily locally available nor can be elicited from study participants. With this in mind, promotion of “equal” participation and contribution of all actors in clinical trials is, ultimately, challenging. Despite the relevance of community participation in health interventions, few studies to date have called into question *who* the so-called “decision-making participants” are, and *how* they are selected; nor, *how* to engage in community participation in order to co-design and co-implement clinical trials (15–18).

To address these questions, “community” should be defined and, its members and/or stakeholders identified. Commonly, a community is said to constitute of a heterogeneous and changeable agency, often sharing geographical locations or settings, common interests, ecology, locality, and/or social ties and system (19, 20). Acknowledging the variety of its definitions and forms, this paper focuses on the socio-spatially defined village participating in a malaria elimination cluster-randomized trial in The Gambia as the unit of “community” (21).

In this trial, community participation was included in the planned proposal in order to contextualize trial implementation. We developed and applied a community participatory approach, “Community Lab of Ideas for Health” (CLIH), to identify *who* constitute decision-making participants in the study community and determine *how* to involve communities in a community-based clinical trial. In this paper, we present the methodology and results of our approach and its contribution to harnessing and incorporating community insights into a clinical trial.

## Methods

This study was part of a cluster-randomized trial on “Reactive Household-based Self-administered Treatment against residual malaria Transmission (RHOST)” carried out around Farafenni in the North Bank Region of The Gambia by the Medical Research Council Unit The Gambia (MRCG).

### The Gambia and the MRCG

The Medical Research Council (MRC) is a United Kingdom (UK) government funding agency supporting medical research; the MRC Unit The Gambia (MRCG) is a research unit funded partly by the MRC that has been operating in The Gambia since 1947 to engage in medical research and provide clinical services nationwide. Recently, the MRCG joined the London School of Hygiene & Tropical Medicine (LSHTM), now officially self-referencing as the “MRCG at LSHTM”. The MRCG holds an important position in The Gambia as a provider of health care and services as well as employment to many Gambians working at the MRCG (22). Moreover, it maintains a unique presence in the country, with a few regional research sites, on-going circulation of international researchers, and a body of local fieldworkers and nurses. The MRCG's presence and influence on the population is palpable, especially set against the comparably weak existing national health care system, and its longstanding and successful work in the country (e.g. bed-net studies, childhood vaccinations) from which stems its reputation as an institution that “helps poor people” and “maintains good health” in The Gambia (22). At the same time, there are also some rumors claiming that MRCG steals and sells blood (23), with some people believing the MRCG enriches itself with blood transactions (24). Notwithstanding, with MRCG's dedication and therefore positive health outcome of population, people are willing to be “joining MRC(G)” (24) for its interventions.

Historically, The Gambia has been economically and politically influenced by and dependent on the UK since the colonial period (25, 26). Over time, the country's dependent key sectors – including the health care sector – invited international, external governments and non-governmental agencies to engage in research and development. Likewise, The Gambia's compact size offered a fertile landscape for scientific activities (26). The MRCG's research, focusing on health issues related to poverty (e.g. malaria, tuberculosis, and malnutrition), aligns with the government's development agenda, which continues to create favorable government policies ensuring the continuity of MRCG research.

Set to such a backdrop, the history, relationships, power, trust and mistrust of the research institution are invoked (27) and this needs to be well acknowledged in any and all MRCG community-based clinical trials.

### Study Site and Population

In the study area, the malaria burden had been declining over the last 15 years (28). Various factors are believed to have contributed to the observed decline, such as the malaria control interventions led by the Ministry of Health and other agencies, including MRCG (28) and an improvement in socio-economic-status (29, 30). Indeed, since the establishment of the MRCG Farafenni field station in 1981, there have been several studies on malaria (23), including those that showed the beneficial effects of insecticide-treated bed-nets on malaria morbidity and mortality. Nevertheless, there is still residual malaria transmission largely due to asymptomatic-carriers who are unlikely to seek care and treatment (31–33). The RHOST (Reactive Household Self-administered Treatment) trial aimed to treat the asymptomatic reservoir and evaluate the effect of reactive treatment on malaria transmission — i.e. full antimalarial (Dihydroartemisinin-Piperaquine) treatment to all residents of a compound with a confirmed clinical malaria case (21). In total, 34 villages were purposely identified based on malaria prevalence (<5%) and randomly assigned to either the intervention or the control arm (21).

The “communities” in this paper represent the 17 villages that were to receive the intervention. The population of these villages was comprised of community-members of different ethnicities – mainly Mandinka, Fula and Wolof, and minority groups of Bambara, Turka and Tilibonka — each with their own language. Most of the village population were Muslim and farmers. English literacy in these villages was low. Almost all villages were located far from the main road and health facilities. Among these 17 villages, 10 had their own village health worker (VHW), a community-member trained by the government, that acts as a link to the Gambian primary healthcare system, namely for health promotion, prevention measures, treatment of minor ailments, and case referral (34).

### Transdisciplinary Trial Design

In order to adapt the trial to the local settings, a trial research team consisting of experts in epidemiology, health economy, health systems, and medical anthropology worked together on the development and implementation of the trial to encourage a systemic and holistic approach (35) to malaria elimination. This transdisciplinary approach was designed to be dialogical, iterative and flexible, enabling tailoring and adaptation of strategies in the midst of the trial intervention process: (i) phase-0 accounted for preparation and development of the implementation strategies; (ii) phase-1 for pilot implementation and fine-tuning of the strategies; and (iii) phase-2 for adaptation of strategies and implementation. Flexible design and iterative exchange within the trial team and with community-members were essential to respond to the complexities of this particular intervention.

The intervention was complex, most of all because treatment had to be self-administered, relying on potentially malaria-infected but otherwise healthy household members to participate. Moreover, the medicine-dosing schedule and distribution were complex, and there was ambiguity as to the appropriate treatment-unit to be targeted. In an effort to overcome these bottlenecks, the medical anthropology team engaged in communicating and co-developing context-specific implementation strategies together with communities, which developed a community participatory approach “Community Lab of Ideas for Health (CLIH)”.

The field researchers for the CLIH consisted of external and local researchers with extensive research and implementation experience in Africa, Latin America and Southeast Asia with a range of disciplinary expertise in anthropology, ethnographic and implementation research, developmental implementation, and health promotion. Local fieldworkers were proficient in all major languages in the study area (Mandinka, Fula, and Wolof) and had high cultural sensitivity, social interaction skills, and previous experience working in social science studies within clinical trials. Additionally, one of the external researchers (first-author) was fluent in Wolof, a language understood by most community-members regardless of their ethnicities. This diverse yet complimentary workforce was key in uncovering local realities and perspectives that have otherwise been overlooked.

### CLIH Conceptual Framework

Our community participatory approach was inspired and guided by Paulo Freire's dialogical approach, which emphasizes “generative themes” that investigate people's praxis (people's thinking about reality and action upon reality) and stimulate their awareness and critical understanding of their reality as interacting constituent elements of the whole (36). The basis of our approach was further influenced by (i) Mark Nichter's formative research and (ii) Socializing Evidence for Participatory Action (SEPA) by Tropical Disease Research Center (CIET, for its acronym in Spanish). Nichter's formative research involves communities in several steps including designing, implementing, and monitoring and evaluating the intervention, and developing health messages (37). CIET's SEPA partners with communities and repeats cycles of research and action to identify and solve development challenges (38–40). In our study, we focused on understanding communities through ethnographic work (which helped investigating “generative themes”) and by presenting this collected information back to communities in a more organized, systematized, and developed manner in order to situate the discussion in the sphere of their local realities and interests (36). We further incorporated the *Kaizen* concept, which is an iterative and accumulative process of steps and innovation necessary for the adjustment and improvement of trial implementation, achieved by constantly generating questions until finding the root cause of problems (41, 42). We ultimately named our approach the “Community Lab of Ideas for Health” after the concept of the *Living-Lab*, which is a user-centered, open innovation environment implemented in research-field settings that facilitates collaboration among involved actors, e.g. researchers and communities (43).

All of these concepts share an emphasis on interactive and iterative processes that generate mutual understanding and strive to reinforce respect and trust between the communities and the researchers, to strengthen creativity and to draw genuine perspectives into discussions (36, 42, 43).

### CLIH Process and Steps

**Figure 1:**
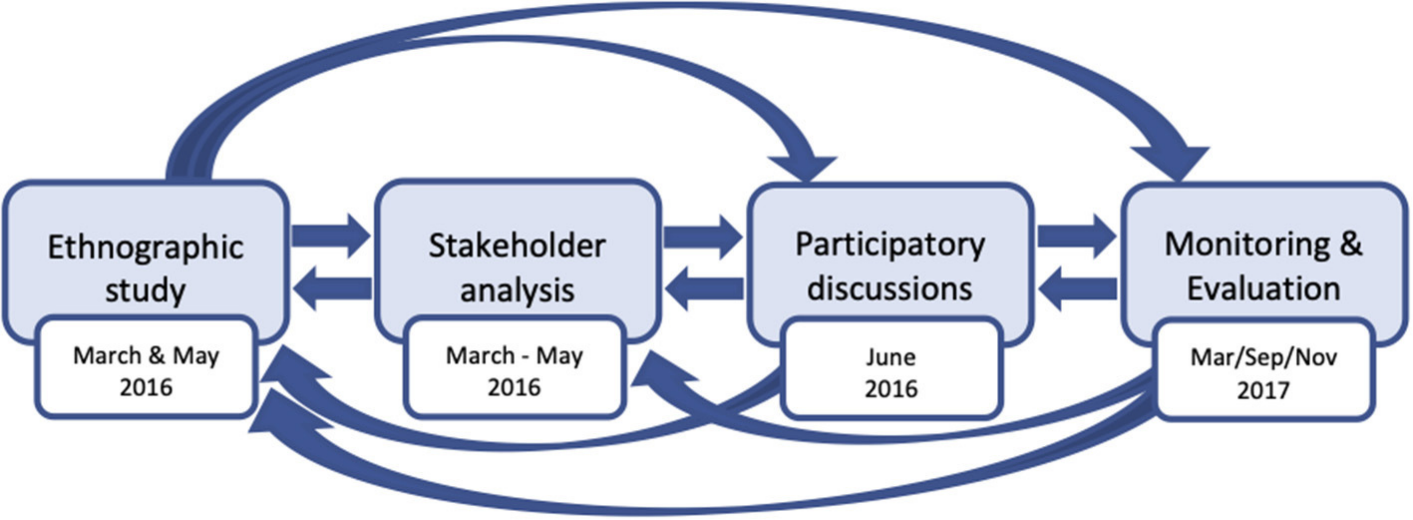
Cyclical process of the community participatory approach.

The key methodologies in our participatory approach consisted of ethnographic study, stakeholder analysis, participatory-discussion, and qualitative monitoring and evaluation, all of which are detailed below. Paramount to this approach is that each element builds upon the other elements, making it a cyclical process (Figure 1).

Researchers and local fieldworkers collected data by visiting or staying in the 17 intervention-villages to observe and build relationships with the communities. The research team developed preliminary interview and discussion guides as well as the fieldwork itinerary prior to visiting the villages; thereafter, the fieldworkers communicated with the *Alkalo* (village head) and VHW in each village who then informed the community-members about the project. In addition to the CLIH, for the trial operation, we conducted quantitative questionnaires (base-line and end-line) and adherence (case) studies, to both inform and validate qualitative findings (results presented elsewhere).

#### Ethnographic Study

##### Rationale

The ethnographic study was the entry point of interaction between the communities and the research team. We aimed to understand villages' socio-cultural-economic environment as well as people's illness perceptions and health-seeking-behavior, and moreover, to identify key-informants relevant for trial implementation to facilitate the construction of a clinical trial addressing the actual needs of communities (44).

##### Data Collection and Sampling

We conducted in-depth interviews (IDIs), informal conversations and engaged in participant observation in 17 villages. In each village, we first introduced ourselves to the *Alkalo* by following the local tradition of presenting him with Kola nuts. After the introductions, we carried out snowball sampling, then proceeded to interview: e.g. compound and/or household heads; mothers and/or caretakers; people who recently had malaria; VHWs; traditional birth attendants (TBAs); traditional healers; Imams; and members of village committees (e.g. development committees, women's groups, youth groups). We conducted 186 IDIs, numerous informal conversations and continuously engaged in participant observation. During participant observation, we took note of hierarchies among household/compound members, neighbors, visitors, youth and the elderly, available malaria protective measures, day-to-day socio-economic activities, and mobility.

#### Stakeholder Analysis

##### Rationale

After the ethnographic study, we recruited key-informants through stakeholder analysis to participate in the study (e.g. partake in participatory-discussions and trial implementation). Stakeholder analysis is a process of identifying individuals to be involved in a project by delving into their behavior, interests, networks, and influence in their community, and thus assesses the effect of their potential participation and influence on the project, as well as determining why and how these individuals should be involved (45, 46). We chose to use stakeholder analysis to sharpen co-development and co-facilitation of implementation strategies with identified key-informants who represent a wide spectrum of community-members.

##### Data Collection and Sampling

We carried out SWOT (Strengths, Weaknesses, Opportunity, and Threat) analysis with 95 IDIs (out of the total of 186 IDIs) to focus on key issues that could affect the strategies and success of the implementation process (47).

#### Participatory-Discussions

##### Rationale

The participatory-discussions allowed us to validate ethnographic results, as well as for the researchers and communities to dialogue, discuss the relevance of the study, share ideas about solutions to issues around malaria and trial implementation, and co-develop implementation strategies. The research team introduced the aim and relevance of the trial to the communities, which was “to reduce the problem of malaria in the area”. At this point, we discussed what was relevant to communities in relation to the trial and why they would or would not want to participate. Once the relevance of the trial and, likewise, community participation was determined, the research team and the key-informants brainstormed solutions to tackle challenges that would potentially affect the trial implementation process.

##### Data Collection and Sampling

The fieldworkers contacted pre-identified key-informants individually and asked them to participate in the participatory-discussions. Once the key-informants agreed to voluntarily take part, we consulted with the village head *Alkalos* and VHWs to schedule the participatory-discussions. We carried out a total of 10 discussions in 17 villages. In order to facilitate participation in the discussions, researchers and communities considered: (i) the timing – before the rainy season so as not to interfere with the work intensive farming season; and (ii) the language – we used one main language, however, multiple languages were used interchangeably at participants' convenience. To have dynamic discussions, we put small villages together (1 discussion for 2 villages) that: (i) were located close to each other; and (ii) shared relatives and had an amicable relationship. *Alkalos* did not take part in these participatory-discussions as their position in the community could have biased participants' responses. Nevertheless, we included them in the trial implementation and monitoring and evaluation processes, and we kept an open line of communication with them to consult and inform them of the project.

#### Monitoring and Evaluation

##### Rationale

We conducted qualitative monitoring and evaluation (M&E) articulating and adapting Realist Evaluation and Most Significant Change (MSC) methodologies to adapt and improve trial implementation strategies. Realist Evaluation is a theory-driven evaluation process that asks “what works for whom in what circumstances and in what respects, and how” [(48): p.4]. MSC is a narrative-based approach designed to identify and analyze qualitative changes as experienced by those involved (49).

##### Data Collection and Sampling

The fieldworkers made follow-up calls twice a month to VHWs who acted as the main implementer of the trial (see results) to promptly detect challenges, rumors, and complaints about the trial so that the trial team could immediately respond and improve the implementation. In addition, we conducted three focused M&E sessions. After phase-1 pilot implementation, we assessed if and how the strategies worked, evaluated VHWs performance, determined challenges in implementation, and exchanged ideas with the communities on how to improve trial implementation for phase-2. During phase-2 implementation, we examined if and how the adapted implementation strategies worked. At the end of phase-2, we evaluated the implementation as a whole and took note of how people experienced it. In total, we conducted 67 IDIs and 18 participatory-discussions, and continuously engaged in participant observation involving informal conversations with VHWs, *Alkalos*, key-informants, compound heads, and caretakers (women) of malaria patients.

### Data Analysis

**Figure 2:**
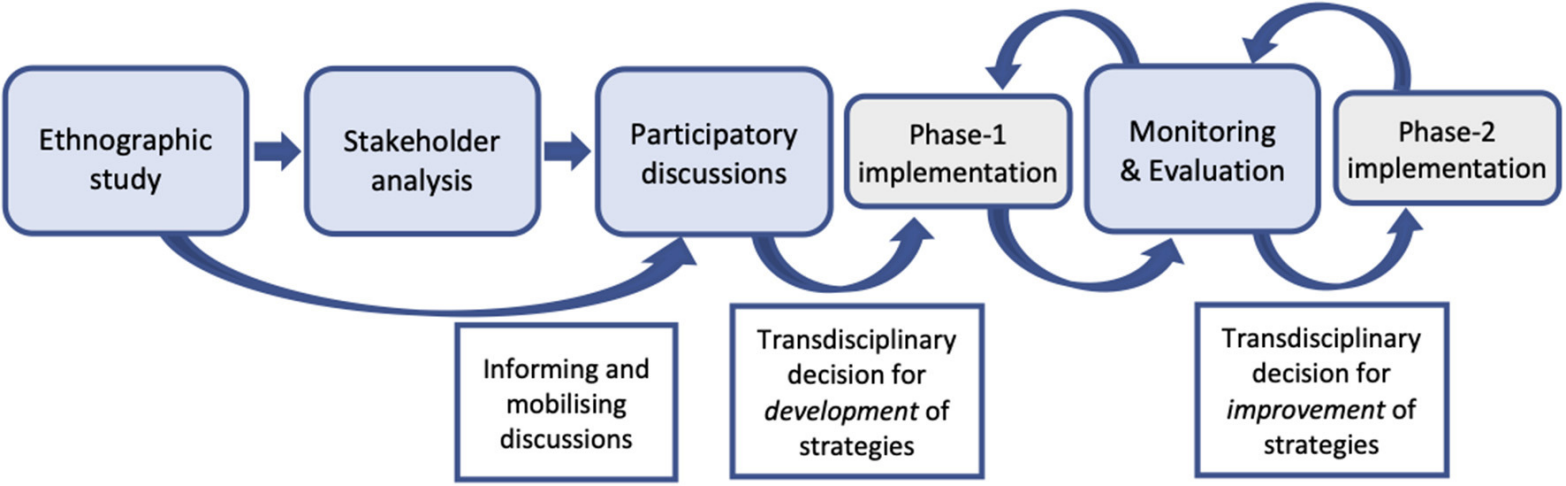
Analysis and reflection of findings to iterative process.

We analyzed collected data every day to adapt the topic guides and determine the next steps of the research process. Data analysis followed an iterative process, and results were shared among the trial transdisciplinary team to further triangulate and analyze the feasibility of implementation strategies (Figure 2).

#### Interviews, Observation, Conversations, and Discussions

The fieldworkers transcribed interviews, verbatim, in English during and after each phase of fieldwork. Researchers took detailed notes of observations, conversations, and discussions, and transferred those notes into digital form immediately after data collection every day. We used NVivo qualitative software to code transcribed interviews and notes both inductively (i.e. generate new theory from data) and deductively (i.e. test an existing theory through observations) for in-depth analysis. For coding, we used themes such as “health-seeking-behavior”, “malaria literacy”, “economic activities”, and “social structure”, as well as emerging themes on “micro-politics”, the “medical poverty trap”, and “trust (among involved stakeholders)”.

#### Stakeholder Analysis

After the ethnographic study, information on the interviewees was analyzed and organized daily into a SWOT matrix in order to effectively identify key-informants for project planning and strategy formulation processes (47). We further ranked each interviewee's information from zero (non-significant) to three (most-significant). Based on our ethnographic results, we considered potential key-informants who could participate and lead their community in the implementation process, showed willingness to collaborate, and, most importantly, were trusted by the community. These community capacities (e.g. leadership and communication skills) predominantly identified in stakeholder analysis were key assets to the implementation process allowing the researchers to rely on local expertise to help shape the intervention. We excluded individuals who clearly expressed unwillingness to participate and/or who would be absent from villages.

### Ethical Considerations

Ethics approval for this study was obtained from the Gambian Government/MRC Joint Ethics Committee (SCC1438v2) and the Institutional Review Board of the Institute of Tropical Medicine, Antwerp, Belgium (1046/15). The research's aim and respondents' rights as well as confidentiality were explained and oral consent was obtained before each interview. Oral consent was preferred due to the high rate of illiteracy among the study populations and to avoid sowing mistrust in communities by obliging signatures. Individual data was stored anonymously in a protected device with a password and was only accessible by the research team. There was no payment involved with interviewees or participants; however, possible indirect benefits to the community, such as the greater malaria elimination efforts and potential benefits, were explained before each interview.

## Results

### Relevant Insights for Trial Contextualization

#### Society, Economy, and Micro-Politics

The most influential and respected position in the village is the *Alkalo*, a role traditionally inherited by males from the lineage of the village founder. A village is organized into compounds consisting of nuclear and extended families, and led by the compound head, who is usually male and has the final say in compound decisions. A compound consists of several households and is the unit of residence, production, and health management. A household is composed of a (male) head-of-household, his wife (wives), and their children. Most people in the village engage in cash-crop and subsistence farming, and herding. During the busy farming season (rainy season), seasonal workers can be hired and will stay in the compound for several months.

#### Asymptomatic Malaria and Protection

To most community-members, it was common knowledge that some diseases, both biomedical and folk illnesses, such as yellow fever, worms, lung tuberculosis, *jinn* (spirits) invoked illness, and malaria, can be “hidden” in the body. This condition of hidden illness was understood as a disease being present in the body, but not affecting one's appetite nor restricting his/her ability to carry out daily activities (see also Jaiteh et al., 2019) (50). Notably, people generally decline taking medicine when feeling healthy, but will accept treatment when the disease is “hidden” in the body, so as to prevent transmission of the hidden malady to family and neighbors that could hinder work requirements. Therefore, in relation to malaria, incentives for taking medicine are to protect the family (i.e. immediate household family as well as extended compound family) from transmission and to minimize economic loss due to health seeking. Some participants perceived the protection provided by antimalarial medicine to work similarly to vaccinations, lasting for a few months or more. With regard to malaria prevention, respondents often emphasized the need to sleep under bed-nets, and participate in *Set-Setal* – the practice of cleaning in/around one's compound and the village-organized cleaning of public spaces (e.g. wells and mosques).

#### Malaria Burden and the Medical Poverty Trap

We explored communities' perspectives, in-depth, on how and why malaria impacts compound and village life. In the study villages, where malaria incidence is declining over the past 15 years, community-members no longer perceived malaria as a fatal disease, unless we explicitly prompted or they realized that the trial focused on malaria. However, malaria continues to pose a serious threat when set to the backdrop of participants' persistent economic vulnerability. The malaria season coincides with the rainy season when the agricultural workload is heavy, cash availability is low, and road conditions are so poor that easy access to health services is close-to-impossible.

“*There is difficulty if you have a patient because from here to the highway is too far. If you don't have means of transport, you must go out and borrow it from another village. It's not easy to ply a donkey-cart on the bad road, so people don't like carrying [a patient]. In the rainy season you cannot have [the donkey-cart] because donkeys will be busy in farm. (IDI, Man, Village-3)”*

In such situations, when someone falls ill, either the patient struggles to reach a health center or caretakers from the village are forced to abandon work obligations to care for him/her. According to the community, these circumstances affect farming productivity and, consequently, income. The impact of malaria can be devastating especially during periods of key agricultural activities, seen also in other studies (51, 52). According to participants, even 2–3 days away from the field have dire consequences (e.g. the loss of 2 out of the approximately 5 bags of peanuts that they normally produce). Moreover, to afford treatment, people are forced to sell stocks of crops, borrow cash with high interest rates, or give away farm materials, which further cripples productivity. Therefore, disease severity does not simply refer to the disease itself, but its associated ramifications that create a vicious economic cycle known as the medical poverty trap (53). For this reason, throughout the study, we placed malaria as just one of the health concerns contributing to the poverty trap. This motivated community-members to participate in the trial because they recognized that the researchers understood the greater health problems they face and that the trial could potentially mitigate their greater health-related socio-economic struggles even while addressing a particular disease (i.e. malaria).

### Improving Implementation Strategies

The following key strategies were co-developed and fine-tuned with communities through several rounds of consultations, discussions, close monitoring and (qualitative) evaluation of the implementation hereby integrating various domains to culminate in a holistic improvement of the implementation strategies:

#### Defining the Intervention Unit

Initially, the trial planned the treatment-unit to be the “household members sharing the sleeping area with the index clinical case”; however, during the ethnographic study, we saw that “treating household members” did not make sense to participants, as the characteristics and definition of what constitutes the household are fluid. In the study area, household members are often understood as the group of people eating from the same cooking pot, but not necessarily living together or sleeping in the same household structure. Moreover, the composition of household members changes when receiving guests or having seasonal workers. Conversely, the compound — comprised of several households — is clearly defined by fences, and represents the unit of residence, production, and health-management. There is frequent interaction between members from different households within a compound, and as such, many participants preferred treating the “compound” as a whole, which includes (extended) family members and seasonal workers in order to better “protect the family”.

“*Treating the whole compound is better, […] because they are all my family. If you treat one house and you didn't treat the other, we are still not safe. (IDI, VHW, Village-1)”*

#### Improving Distribution and Intake of Antimalarial Treatment

**Figure 3:**
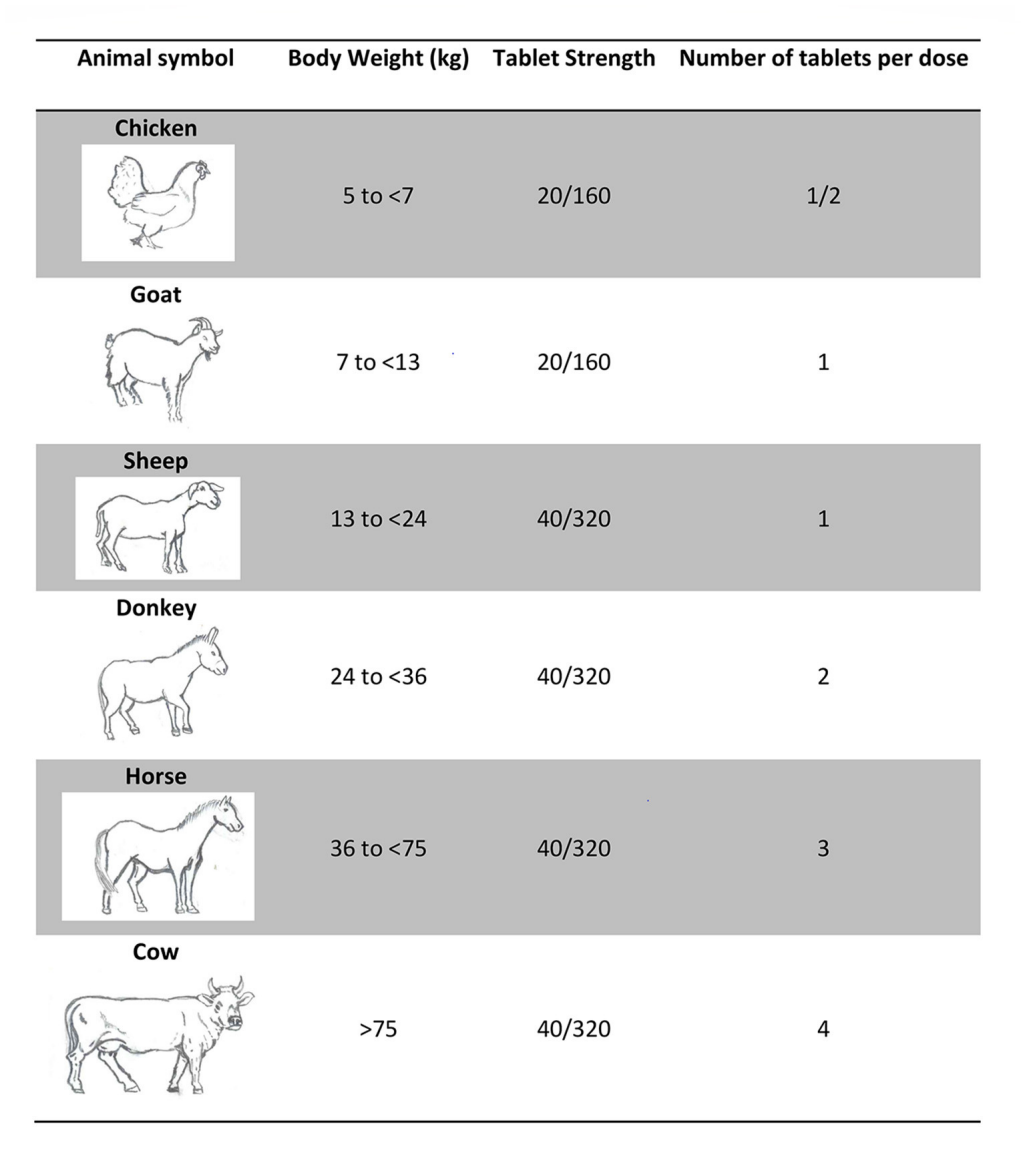
Dihydroartemisinin-Piperaquine (DP) dosage and animal symbols.

Antimalarial treatment distributed to compound members of a confirmed malaria patient was Dihydroartemisinin-Piperaquine (DP), the second-line antimalarial treatment in The Gambia. DP doses are based on body weight and categorized into six treatment groups. While the trial (epidemiology team) weighed the entire population of the study villages prior to its implementation, the low literacy rate posed a challenge to self-administered drug delivery (e.g. writing individuals' names on the medicine bag was insufficient). After discussing ideas to recognize different weight categories, key-informants suggested using symbols. Eventually, the use of animal symbols was suggested by key-informants and agreed upon as the best available option to facilitate self-administration of the drug. Animal symbols were easily chosen as the key-informants in all study villages listed the exact same animals that they observe in their daily life: chickens, goats, sheep, donkeys, horses, and cows. Animal symbols were drawn by one of the fieldworkers and approved by community-members (Figure 3). The logbook (created by the epidemiology team) with compound member's names, animal symbols and corresponding DP dosage was handed to VHW.

“*Alhamdulillah (praise be to God), those [animal signs] really helped us a lot. They assisted those who are illiterate so that they were able to identify the animal signs [for DP administration weight categories]. (IDI, Man, Village-16)”*

Another important topic discussed iteratively was distribution and intake of DP by pregnant women during the first trimester. The trial advised these women not to take DP; however, identification of pregnant women posed a delicate question as many women avoid early-stage pregnancy disclosure. Initial discussion between researchers and community-members suggested either privately administering pregnancy tests to suspected pregnant woman or to all women of reproductive age in the compound. However, after further discussions this course of action was deemed inappropriate by key-informants who concluded that such an intervention would pose a potential risk of unwanted disclosure of pregnancy and/or spread rumors.

“*Many women here, their husband has traveled, so if the wife is tested and is pregnant, that is going to cause a lot of problems. Women would not want that to be exposed. We don't want the project to be involved in that kind of scenario. (Discussion, Village-6&7)”*

**Figure 4:**
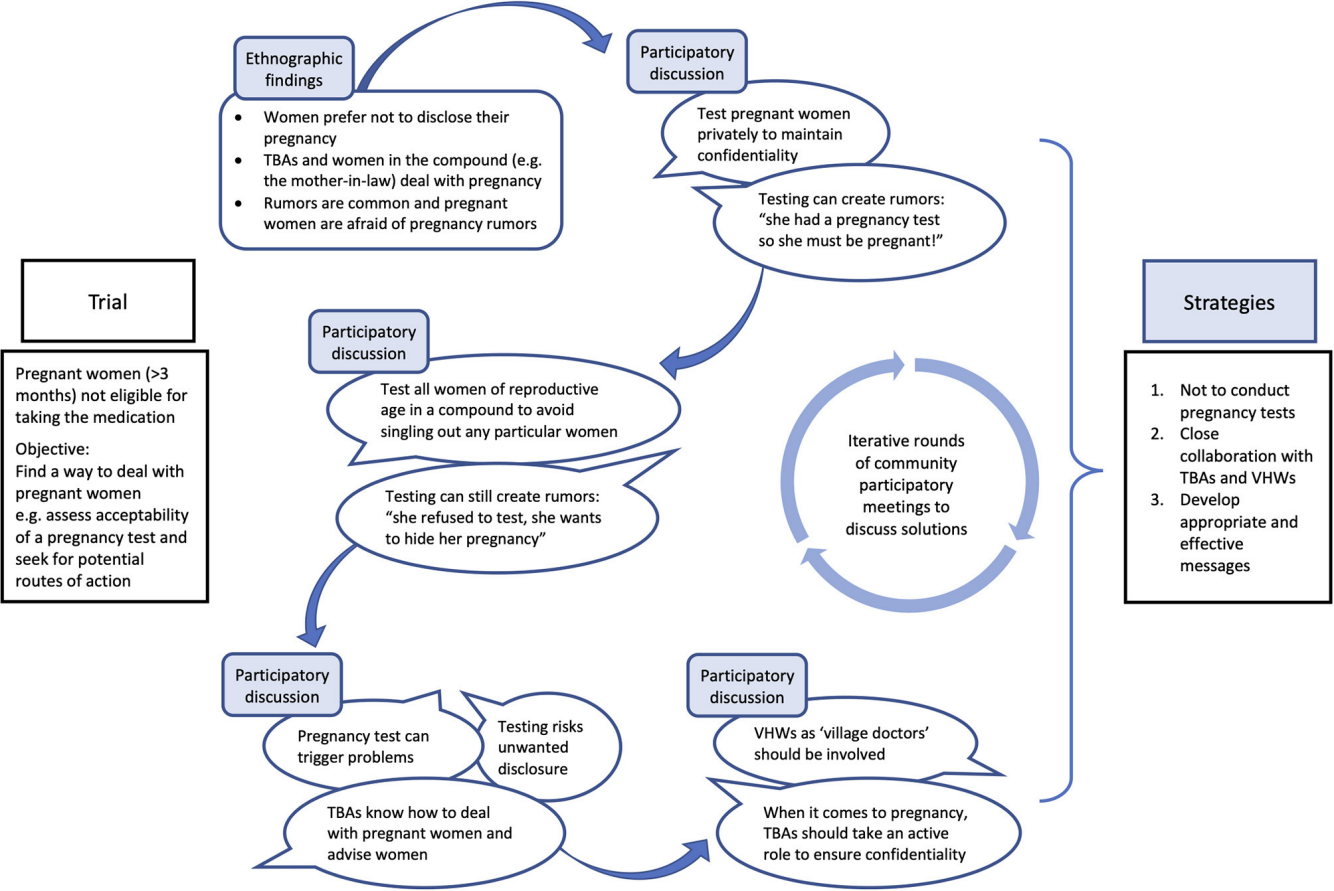
Example: co-development of strategies through iterative dialogues and data triangulation.

Although the topic was ticklish, key-informants discussions also demonstrated the well-established culture of consensus-building in rural Gambia (54). Therefore, despite the divergent emerging opinions (e.g. to test or not to test) and associated tension that arose due to discussion of such delicate issues, after a series of discussions, we agreed to not conduct pregnancy tests but emphasized the need to collaborate with traditional birth attendants (TBAs) and VHWs to advise pregnant women not to take DP in the first trimester of pregnancy (Figure 4).

#### Involving Local Structures in the DP Distribution

The trial initially planned and dispatched MRCG-nurses to prescribe DP to the communities, with the VHW helping with the distribution. This was agreed upon by community-members citing trust in the MRCG and its nurses. For villages without a VHW, the communities appointed one community-member as a Village Collaborator to become a communication link between the trial and the village.

After qualitative monitoring of phase-1 pilot implementation, we noted that people recognized VHW performance had improved (e.g. equipped with diagnostic and treatment tools, they treated patients in a timely manner – upcoming paper by Masunaga et al.) and key-informants insisted on placing DP distribution in the hands of the VHWs. Consequently, in phase-2, VHWs became the main actors in prescribing (i.e. preparing DP medicine bags accordingly to the weights of the compound members) and distributing DP with the support of MRCG-nurses in case of complications that VHWs found challenging to deal with by themselves. However, DP distribution through VHWs presented challenges of its own. Namely, literacy limitations of the VHWs themselves, VHWs' own conflicting work requirements, the absence of compound members likewise maintaining their work requirements, and the sheer number of individuals (sometimes up to 50 individuals) to whom the VHWs had to reach for the distribution proved challenging. To mitigate these obstacles, the communities strongly urged organizing distribution in line with their local structure and micro-politics. Effectively, having DP distributed by the VHW to the compound head who would then distribute DP to the household heads. Thereafter, the household head would have his wife (wives) administer DP to their children. If the compound head was absent, a representative (often the eldest household head or the eldest woman) took over this role.

“*…if you see [VHW] brings medicine here, I am the one who allows him to do that. But if I told him that I don't want to participate in this program, he would not have the courage to bring medicine in the compound. It's like I gave [the VHW] the right to bring medicine in my compound. (IDI, Compound head, Village-17)”*

The trial provided training and a monthly monetary incentive for VHWs (1500 dalasi = ±25EUR) and Village Collaborators (800 dalasi = ±13EUR) at the communities' request. In addition, the trial guaranteed a free supply of rapid diagnostic tests and antimalarial medications to VHWs. VHWs also requested a venue for themselves to see patients. However, based on fieldwork observations of abandoned VHW buildings previously built by an NGO, and awareness that the VHWs' heavy workload would not accommodate them receiving patients, we (researchers and community-members) discussed and together deemed this a fruitless pursuit.

#### Delivering Health Communication

The bases of health messages for the trial implementation process (i.e. trial aim, malaria burden, treatment-unit, asymptomatic-carriers, and DP identification and distribution) were adapted by communities in response to the local contexts. For example, to explain asymptomatic-carriers, we adapted expressions used by community-members: “*Malaria kurango kesso, siitarano mojatokono amafinti” (Mandinka)*, meaning malaria can be hidden in the body without showing. Moreover, we discussed how and when to disseminate the messages with key-informants. Consequently, health messages were disseminated at two levels: community-based and patient-based. Community-based communication consisted of sensitization meetings with key-informants who passed on information by word-of-mouth, and of a public sensitization campaign inviting all community-members in each village to attend the event where health messages were delivered through skit performed by key-informants. Patient-based communication occurred during the VHW-patient interaction explaining DP dosage and schedule and informing a compound visit for DP distribution.

### CLIH Approach and Trust Building

Generative dialogue and working along the existing community structure increased people's acceptance, confidence, and trust with regard to the trial and its team.

“*I am happy with the way you approach us. […] We discussed how to introduce the project in this village. We gave you some ideas and you also gave us ideas. […] These [strategies] are all steps that we have discussed with you and all that has matched with how people want it to be. (IDI, Man, Village-8)”*

As a result of adhering to the local social system and micro-politics, we successfully involved key-informants who were trusted by people and already considered active contributors to village development (e.g. heads of committees, TBAs and VHWs). Involving these key actors facilitated the smooth execution of the trial implementation. Moreover, working closely with the VHWs and equipping them with testing and treatment tools increased VHW's agency, confidence and preparedness to treat sick people, consequently enhancing trust and motivation of community-members in seeking care at the VHWs.

“*I have enough medicine now. If a soldier is armed and sent to a battlefield, he should be prepared to fight. (IDI, VHW, Village-7)”*

In addition, people tended to more readily accept programs and treatments that the MRCG offered in the communities due to its longstanding presence and trust in the country and the observed reduction in malaria that many attributed to the MRCG's interventions.

“*Since we started this project, throughout this malaria season, fortunately we have no malaria case. I believe that the advice you gave us from the start of the project, people maintained it. That's why I see we don't have any malaria case, because we protect ourselves from malaria. (ID, VC, Village-15)”*

“*This project, Alhamdulillah, is working well in this village. Everyone in the village appreciated it. That's why when MRC(G) is calling, the whole village will respond to it and welcome you. (IDI, VC, Village-16)”*

“*The MRC(G) is our culture here. They have been here for a long time since we were very young. (…) [This village] and the MRC(G) have been together for a long time. (IDI, Man, Village-11)”*

Interestingly, above quotes are all from villages where recorded no malaria case during the trial period, meaning that there was no MRCG intervention (i.e. DP distribution, MRCG nurse supervision). This highlights MRCG's distinct presence in local communities likely influencing people's responses in favor of the MRCG. For another instance, bed-net use to prevent malaria transmission was emphasized (e.g. be inside the bed-net around 8pm) by the community-members at the beginning of our research. However, once confidence between the researchers and communities was built, respondents later revealed:

“*We know the risk [of malaria], but we still prefer chatting outside at night because we are fond of it. (Discussion, Village-4)”*

## Discussion

Today, trial researchers and implementators are becoming more aware of the necessity to remain responsive to local contexts, many of whom are attempting to carry out some form or another of formative research and/or community engagement. However, designing complex interventions requires going beyond cursory formative or community-based research. There are a handful of interventions in the field of malaria that have successfully developed a strong formative and community-based approach. For instance, a three-year intervention in Nigeria to improve seasonal malaria chemoprevention employed a solid formative research component and successfully identified opportunities and challenges to design the intervention based upon local contexts and the realities of the health system (55). Likewise, evidence-based interventions to improve quality of care for malaria in Uganda and to support the use of malaria diagnostic tests in Tanzania successfully showed how iterative, well-structured, and evidence-based implementation research facilitated the successful design of complex interventions (56–59). In addition to employing sound formative and implementation research as seen in these laudable examples of trial implementation, the CLIH goes on to place marked emphasis on the co-creation of implementation strategies, allowing this process to identify the local idiosyncrasies that generally go unseen by clinical trial research and implementations.

With CLIH, continuous dialogue with communities enabled a merging of the needs of the study with those of the communities, and created common goals to reduce malaria infection and related economic struggles that often leads to the medical poverty trap (53). This joint negotiation of the goal is important in sustaining willingness to commit achieving that goal (60). Moreover, the generative negotiation process enabled us to (i) incorporate a wide spectrum of participants and perspectives into discussions, (ii) critically question the so-called “community-based knowledge” that is often presumed to be the mend-all for trial challenges (1, 4), and (iii) build confidence between stakeholders which led to high levels of acceptance and uptake of the trial intervention.

### Why CLIH

Firstly, the CLIH process involving ethnographic research followed by stakeholder analysis allowed us to interact with a range of community-members, many of whom did not necessarily wield power or influence, and therefore gave us access to heterogeneous range of perspectives. Effectively, this process allowed us to transcend community-participatory studies limited to willing participants (61, 62) or place sole emphasis on the “marginalized voice” (7) but reach a variety of community actors and incorporate a range of political and social dynamics into our research. As Cornwall (63) warns, “giving voice” to limited representation entails a danger of reproducing existing inequality. In the case of RHOST, after engaging with a diverse range of community-members, we came to understand the well-structured society and the pivotal manner of respecting local micro-politics.

Secondly, these diverse and heterogeneous perspectives formulated different narratives of community realities, in which we could siphon genuine local knowledge from historically imbedded project-derived knowledge. One of the main challenges many community-based projects face is the articulation of knowledge that communities present as their own, but which is mostly acquired from previous experience with global health interventions (64). People internalize such “project-knowledge” (i.e. a body of knowledge, values and priorities, ways of performing, dynamics and ideologies related to malaria or other health projects) that becomes packaged in the “project-box” (i.e. internalized project-knowledge delineated by prevailing power-disparities) over years of continuous health interventions in the study area. A classic example is often seen in the knee-jerk community responses to questions concerning malaria prevention: “use a bed-net”; “create awareness”; “ensure a clean environment” where, following Freire's alienation theory (36), “words are emptied of their local concreteness and become a hollow, alienated, and alienating verbosity” (p.71). In our study, the ill-conceived request to build (another) VHW patient-house illustrated how projects/researchers can misinterpret project-knowledge as local knowledge/solutions. We observed how such a community-generated “solution” was not actually founded upon the local realities and therefore acknowledged as not sustainable by the communities themselves. Here Freire's “generative themes” come into play as they investigate people's realities in order to become aware of the influences over what constitute realities (36). Moreover, there are methodological biases that influence informants' views or responses (e.g. informants provide answers they believe researchers want to hear, data collectors influence data due to their morals, beliefs, experiences or logistical limitations) (65–67) that can be erroneously interpreted as authentic local knowledge. Law (68) calls it “performative understanding of methods” that treat knowledge practices as “performative” where knowledge is created and realities (that are not real outside of a particular realm – e.g. a clinical trial) that fit with created knowledge are generated. In this sense, local solutions or local needs tend to be stereotyped recipes reproduced and shaped by perceptions of what a project plans to implement (12) over years of health interventions that may vary from one intervention, site, and topic to another. In the RHOST study villages, people had been exposed to a number of projects implemented by the Ministry of Health, NGOs, and the MRCG, further reinforcing, though albeit unintentionally, project-knowledge, and potentially biasing the process and outcomes of research (22, 69). It is a “false belief” to think that communities involved in a project are “equal” partners (66) in navigating this process. The CLIH endeavored to level the playing field by creating generative dialogue that encouraged both researchers and participants to venture out of the “project-box” and that would engage local and personal realities in problem-solving mechanisms. According to Boyd and Goldenburg (70), creative solutions are often hiding within the existing environment “inside-the-box” that is more community-based, locally-oriented, and embedded in people's life. In order to reach inside-the-box, we generated questions until we succeeded in engaging in communities' realities (36, 41) as seen in the joint-creation of animal symbols to address the challenges of DP self-administration, and in the continuous dialogue to reach consensus on how to handle DP administration during early pregnancy. These generative dialogues were key to successfully translating the more abstract themes of a clinical trial (i.e. treating asymptomatic malaria) into concrete and personalized issues that people could identify with (i.e. protection from *hidden* disease and associated economic pitfalls thereof) and in which communities could see the relevance of their participation in the trial implementation process.

Lastly, this generative dialogue process built mutual understanding, respect and trust between the communities and the researchers, and therefore succeeded in eliciting people's genuine opinions (36, 42, 43) to co-create the trial strategies. Trust is often mentioned as a crucial element in global health, yet it is usually an undermined concept in health program planning (71), reflecting unequal power dynamics in decision-making process. For the RHOST, the trust and confidence built up by the CLIH process served as the backbone of the trial's successful implementation. This confidence was not created overnight, but was a product of the continuous interactions, where communities ultimately entrusted researchers to reflect their voice in the trial and researchers largely entrusted trial implementation to the communities.

Sustaining communities' motivation is vital to malaria elimination efforts (72). However, we found it difficult to maintain community enthusiasm for and participation in an intervention that was not considered a priority in participants' everyday life. Therefore, instead of attempting to artificially sustain motivation, we nurtured interest in participation by engaging in topics that effected the community (e.g. reduce health-related burdens), by exchanging locally-oriented ideas, by reinforcing pre-existing structures (e.g. working with VHWs), by complying with local micro-politics, and by earnestly building up trust. This painstaking process bridged the gap between research and implementation and resulted in an unprecedented level of intervention uptake (92%) (73) despite the dual challenges of self-administered treatment for asymptomatic malaria-infected carriers. Additionally, the essential outcome of CLIH (i.e. RHOST implementation strategies) was applied to additional villages (in South Bank Region) the following year. Without undertaking the entire process of research and community engagement, the trial confirmed high uptake of the intervention by following the same implementation strategies (74). This suggests the scalability of the strategies developed through key findings from the CLIH. We are positive that the CLIH iterative generative process can be applied in various settings for a range of topics and enjoy similar success.

### Limitations

We acknowledge that there were inevitable disparities (i.e. knowledge, power, expertise) between communities and the research team. In an effort to minimize such disparities, the CLIH required resource (time and human) investment. The researchers spent vast amount of time visiting and staying in the villages. The researchers and fieldworkers had to remain flexible (in time, action, thinking) and respectful (to the local way of living) to gain people's trust. Especially it required human resource who, not only spoke local languages, but also understood unspoken customs (sometimes in a very subtle and discreet manner).

## Data Availability Statement

The datasets presented in this article are not readily available because study participants did not consent to have their full transcripts made publicly available. However, the NVivo database with excerpts of the transcripts relevant to the study is available from the corresponding author on reasonable request. Requests to access the datasets should be directed to Yoriko Masunaga, ymasunaga@itg.be.

## Ethics Statement

The studies involving human participants were reviewed and approved by the Gambian Government/MRC Joint Ethics Committee (SCC1438v2) and the Institutional Review Board of the Institute of Tropical Medicine, Antwerp, Belgium (1046/15). Written informed consent for participation was not required for this study in accordance with the national legislation and the institutional requirements.

## Author Contributions

YM and JM conceptualized the community participatory approach CLIH and implemented its process, collected and analyzed the data, and drafted the first phase of manuscript. KP and JM designed the anthropological work-package of the trial. YM wrote the manuscript and designed the figures. FJ contributed in part of data collection and analysis. EM translated interviews and discussions, transcribed data, and helped interpret the data. KP supervised the data collection and the process of manuscript writing. CN-S, DV, RG, KP, and JM guided intellectual debate on the topic. JB, JO, and UD'A added insights to the manuscript. All authors reviewed and approved the paper.

## Conflict of Interest

The authors declare that the research was conducted in the absence of any commercial or financial relationships that could be construed as a potential conflict of interest.
